# Supplementary material for: Innovative Fluorinated Polyimides with Superior Thermal, Mechanical, and Dielectric Properties for Advanced Soft Electronics
Source: Polymers (Basel). 2025 Jan 26;17(3):339. doi: 10.3390/polym17030339 (PMC11821238; doi:10.3390/polym17030339)
Supplement: Supplementary file 1 [file polymers-17-00339-s001.zip › polymers-3406652-SI.pdf]

Innovative Fluorinated Polyimides with Superior Thermal, Mechanical, and Dielectric Properties for Advanced Soft Electronics

Supplementary document

Yuwei Chen [1], Yidong Liu [2] and Yonggang Min [1,*]

[1] School of Electromechanical Engineering, Guangdong University of Technology, No. 100 Waihuanxi Road, Guangzhou HEMC, Guangzhou 510006, China; polebear1996@outlook.com
[2] Widerange Flight Engi-neering Science and Applications Center, Institute of Mechanics, Chinese Academy of Sciences, No.15 Beisihuanxi Road, Beijing, China (100190); Guangdong Aerospace Research Academy, Guangzhou (Nan Sha), China
* Correspondence: zlla@foxmail.com (Y.L.); yong686@126.com (Y.M.)

1 The full spectra of FTIR

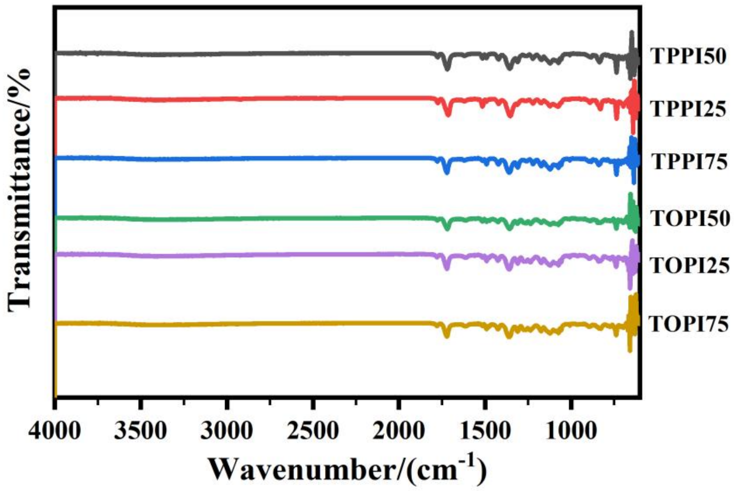

Figure S1 The FTIR spectra of TPIs

2 Discussion of NMR

After a thorough review of relevant literature and conduct of pertinent experiments, it was found that 1H NMR has indeed been employed for the structural characterization of polyimides (PIs) in certain publications. However, the solubility of PIs in most organic solvents is poor. Therefore, tests were conducted using NMR solvents as reported in the literature.

Specifically, TPI was dried in an oven at 60°C for 24 hours. Both the solvent and the TPI film were then weighed. The TPI was immersed in the solvent for 24 hours,

removed, and its surface liquid was wiped off. Both the solvent and the TPI film were weighed again. The results indicated no significant change in weight for either the TPI film or the solvent before and after immersion. This suggests that TPI exhibits poor solubility in liquid NMR solvents, such as deuterated chloroform, which is insufficient for NMR characterization.

Analysis of the relevant NMR-tested literature revealed that most of the studies involved crosslinked PIs, which had low molecular weights prior to crosslinking. This may be attributed to weaker intermolecular forces, thereby enhancing their solubility. In other literature, the PI structure contained groups that improved PI solubility. However, in this study, TPI inherently possessed a high degree of polymerization and molecular weight, with no structural features promoting solubility.

The use of polyamic acid (PAA), the precursor of PI, for NMR characterization was also considered, referencing gel permeation chromatography (GPC) characterization methods. However, a critical issue arises: although PAA and PI have nearly identical molecular weights, the number and distribution of hydrogen atoms in PAA and PI differ significantly before and after imidization (i.e., dehydration condensation). Thus, NMR characterization using PAA would introduce considerable errors.

Solid-state NMR emerged as a potential alternative. However, its inherent limitations and stringent requirements for sample purity necessitate consideration of numerous factors in the structural characterization of TPI.

After careful analysis and consideration, NMR testing was ultimately not selected for this study.
